# Supplementary figures and images for: Drug discovery of small molecules targeting the higher-order hTERT promoter G-quadruplex
Source: PLoS One. 2022 Jun 16;17(6):e0270165. doi: 10.1371/journal.pone.0270165 (PMC9202945; doi:10.1371/journal.pone.0270165)

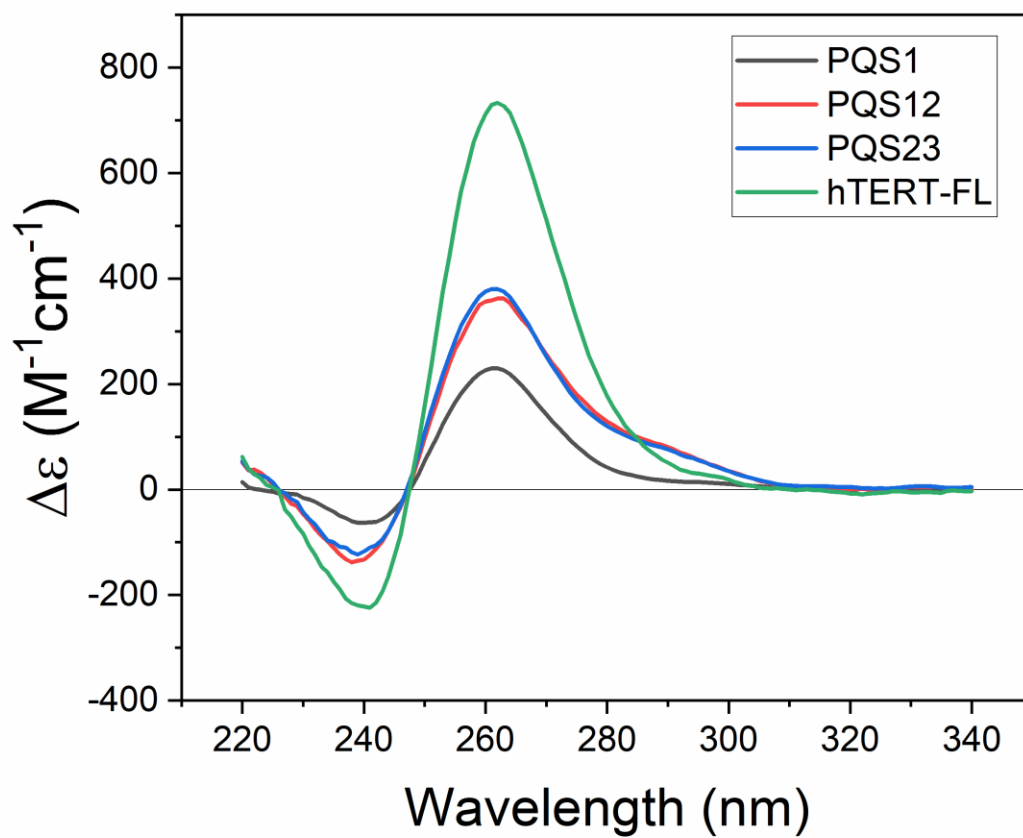

**Figure S1.** CD spectra of PQS1, PQS12, PQS23, and hTERT-FL in BPEK buffer with 100 mM KCl.

Supplement: S1 Fig — (PDF) [file pone.0270165.s001.pdf]
