# Supplementary material for: Drug discovery of small molecules targeting the higher-order hTERT promoter G-quadruplex
Source: PLoS One. 2022 Jun 16;17(6):e0270165. doi: 10.1371/journal.pone.0270165 (PMC9202945; doi:10.1371/journal.pone.0270165)
Supplement: S2 Fig — Red is used to help visualize structural differences between first-generation molecules and derivatives. (PDF) [file pone.0270165.s002.pdf]

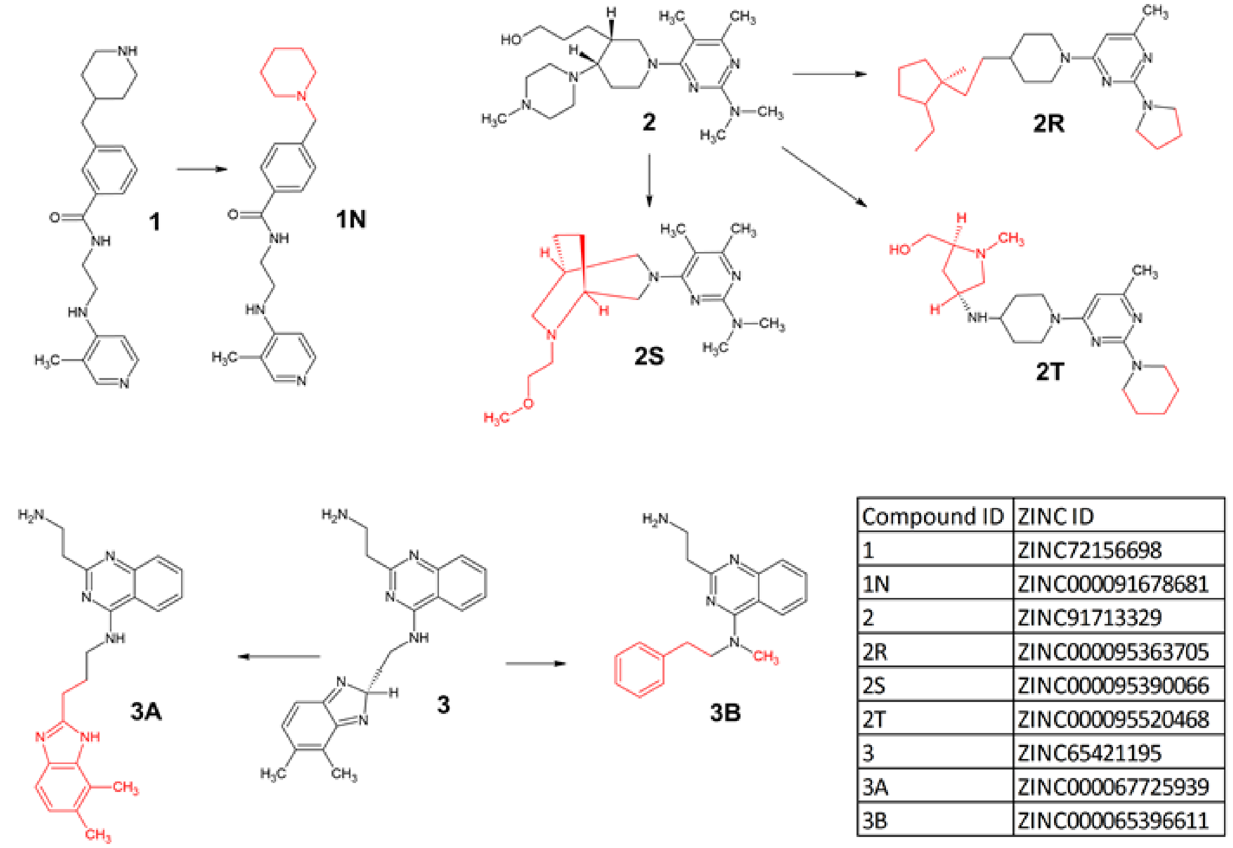

Compound ID	ZINC ID
1	ZINC72156698
1N	ZINC000091678681
2	ZINC91713329
2R	ZINC000095363705
2S	ZINC000095390066
2T	ZINC000095520468
3	ZINC65421195
3A	ZINC000067725939
3B	ZINC000065396611

Figure S2. Structural variations of first-generation compounds (1-3) with their “SAR by catalogue” derivatives and ZINC identifiers. Red is used to help visualize structural differences between first-generation molecules and derivatives.
