# Supplementary material for: Drug discovery of small molecules targeting the higher-order hTERT promoter G-quadruplex
Source: PLoS One. 2022 Jun 16;17(6):e0270165. doi: 10.1371/journal.pone.0270165 (PMC9202945; doi:10.1371/journal.pone.0270165)
Supplement: S3 Fig — Data are plotted as normalized molar ellipticity at 260 nm versus temperature and fit using a sigmoidal Boltzmann function to estimate the Tm (plotted in the lower panel with whiskers representing estimated standard error). (PDF) [file pone.0270165.s003.pdf]

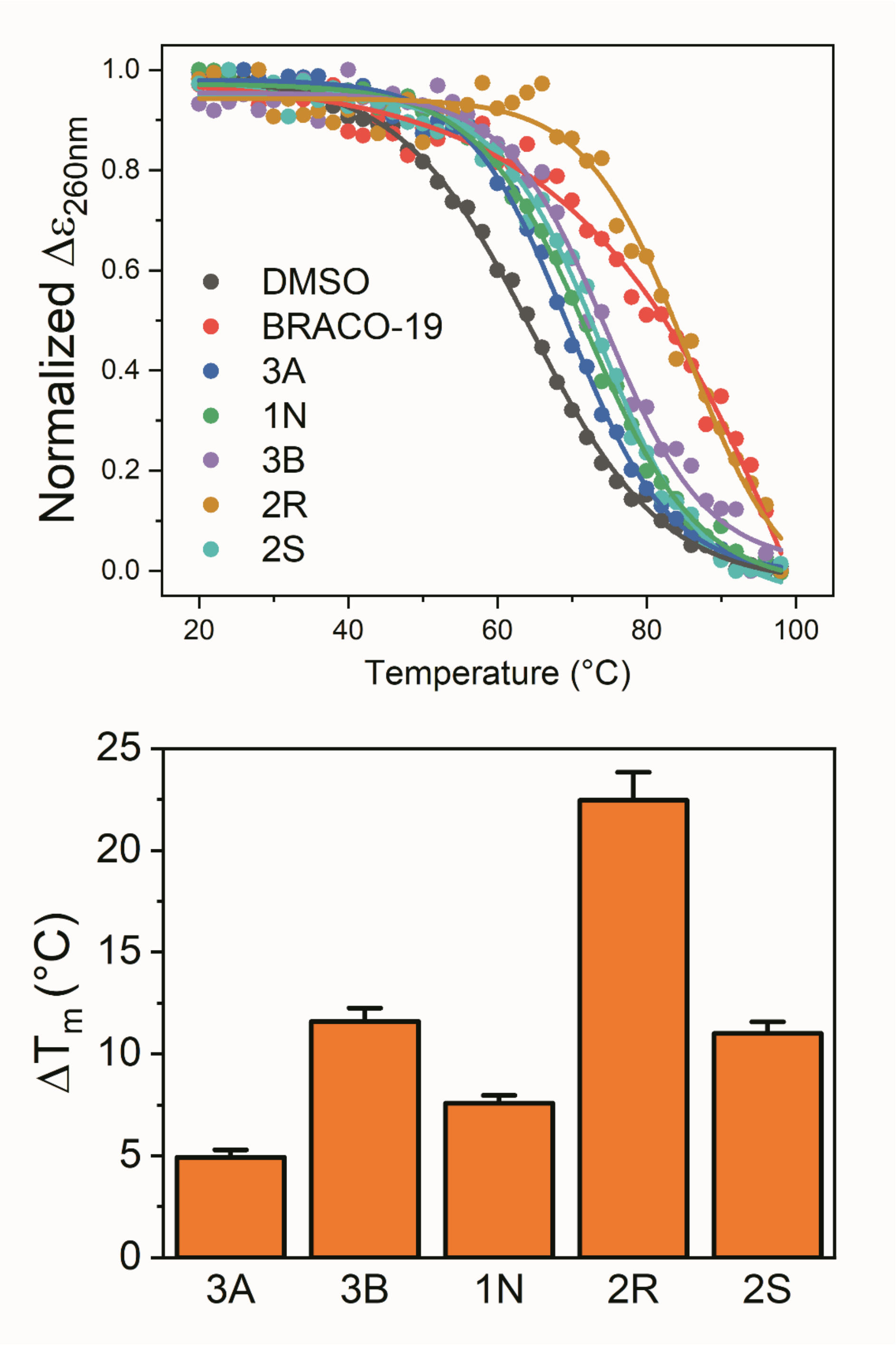

Figure S3. CD melting analysis of hTERT-FL (20 mM KCl) in the presence of BRACO-19 (2.5 μM, positive control), compounds 1N, 2R, 2S, 3A, and 3B (all at 25 μM), or an equivalent volume of DMSO (negative control). Data are plotted as normalized molar ellipticity at 260 nm versus temperature and fit using a sigmoidal Boltzmann function to estimate the T_m. Lower panel shows the ΔT_ms with whiskers representing estimated standard error.
