# Supplementary material for: Drug discovery of small molecules targeting the higher-order hTERT promoter G-quadruplex
Source: PLoS One. 2022 Jun 16;17(6):e0270165. doi: 10.1371/journal.pone.0270165 (PMC9202945; doi:10.1371/journal.pone.0270165)
Supplement: S4 Fig — In each figure, the top panel shows the raw MST traces, and the lower panels show the fit and residuals. The figures show titrations with compounds (A) 3B, (B) 3B1 as an average of quadruplicate measurements, (C) 3B2, (D) 3B3, (E) 3B4 (no reliable fit was obtained for the data), and (F) 3B5. Analysis was done in the “cold fluorescence” mode of the program PALMIST v1.5.8 [60] and fit with a 1-site binding model. Inset in each figure are the Kd values, 68.3% confidence intervals in brackets, and fits (rmsd) in blue. Figures were generated in PALMIST v1.5.8. (PDF) [file pone.0270165.s004.pdf]

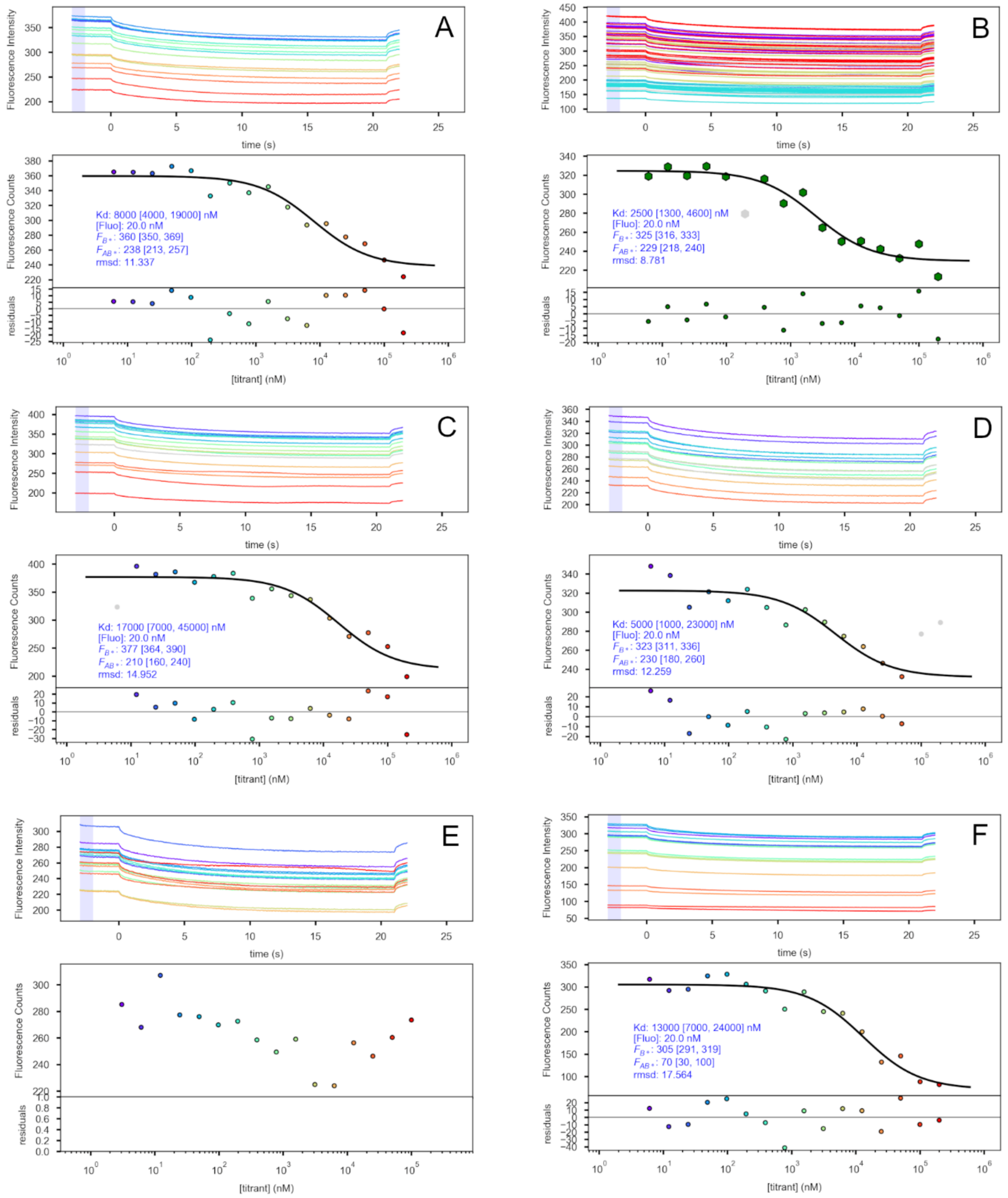

Figure S4. Representative MST titrations for compound 3B and its derivatives with the Cy5-hTERT-FL G-quadruplex sequence. In each figure, the top panel shows the raw MST traces, and the lower panels show the fit and residuals. The figures show titrations with compounds (A) 3B, (B) 3B1 as an average of quadruplicate measurements, (C) 3B2, (D) 3B3, (E) 3B4 (no reliable fit was obtained for the data), and (F) 3B5. Analysis was done in the "cold fluorescence" mode of the program PALMIST v1.5.8 and fit with a 1-site binding model. Inset in each figure are the K_d values, 68.3% confidence intervals in brackets, and fits (rmsd) in blue. Figures were generated in PALMIST v1.5.8.
