# Supplementary material for: Drug discovery of small molecules targeting the higher-order hTERT promoter G-quadruplex
Source: PLoS One. 2022 Jun 16;17(6):e0270165. doi: 10.1371/journal.pone.0270165 (PMC9202945; doi:10.1371/journal.pone.0270165)
Supplement: S5 Fig — (PDF) [file pone.0270165.s005.pdf]

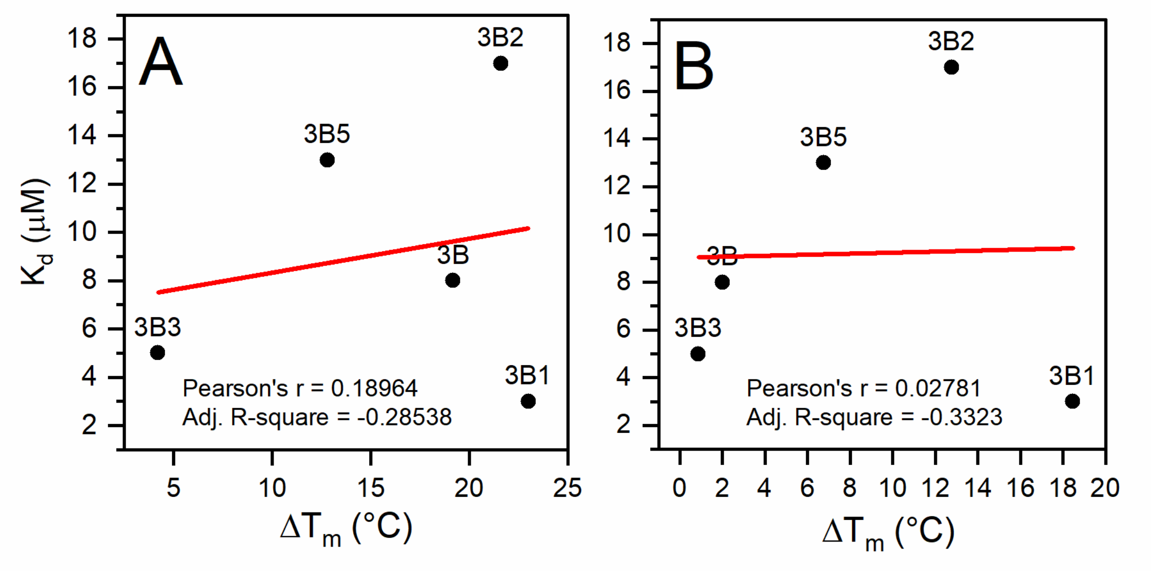

Figure S5. Regression analysis of dissociation constant (K_d) with respect to melt temperature shifts (ΔT_m) for (A) PQS12 and (B) PQS23. Dissociation (K_d) values were derived from the MST data using the hTERT-FL sequence.
