# Supplementary material for: Drug discovery of small molecules targeting the higher-order hTERT promoter G-quadruplex
Source: PLoS One. 2022 Jun 16;17(6):e0270165. doi: 10.1371/journal.pone.0270165 (PMC9202945; doi:10.1371/journal.pone.0270165)
Supplement: S6 Fig — (A) Hydrogen bonding network showing multiple water interactions bridging the 3B1 molecule and residues surrounding the pocket. Hydrophobic ring systems are facing inward toward the G-tetrad column. (B) Space-fill representation of hTERT-FL with 3B1 (green) shown as spherical representation to highlight the size and depth of the binding pocket. (PDF) [file pone.0270165.s006.pdf]

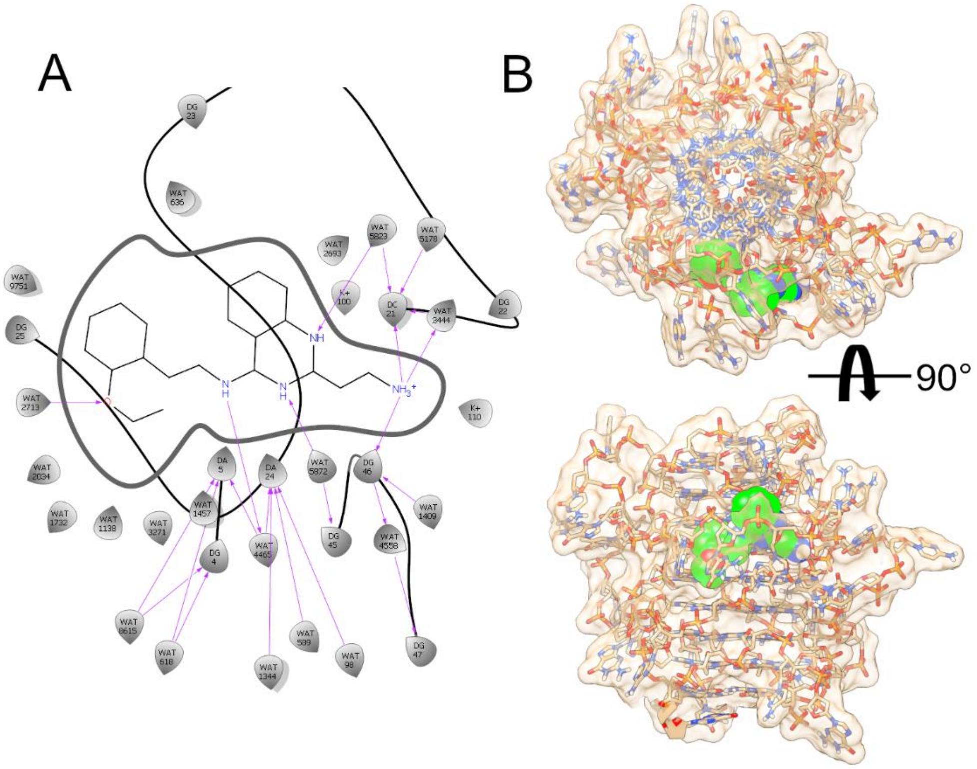

Figure S6. Interaction network and space-fill representation of 3B1 after 5ns of explicit solvent MD simulation. (A) Hydrogen bonding network showing multiple water interactions bridging the 3B1 molecule and residues surrounding the pocket. Hydrophobic ring systems are facing inward toward the G-tetrad column. (B) Space-fill representation of hTERT-FL with 3B1 (green) shown as spherical representation to highlight the size and depth of the binding pocket.
