# Supplementary material for: Drug discovery of small molecules targeting the higher-order hTERT promoter G-quadruplex
Source: PLoS One. 2022 Jun 16;17(6):e0270165. doi: 10.1371/journal.pone.0270165 (PMC9202945; doi:10.1371/journal.pone.0270165)
Supplement: S7 Fig — (A) Hydrogen bonding network showing multiple water interactions, although none are coordinated with residues on the quadruplex. (B) Space-fill representation of hTERT-FL with 3B1 (green) shown as spherical representation. The two orientations emphasize how far the 3B1 ethoxy group (oxygen in red) extends away from the quadruplex. (PDF) [file pone.0270165.s007.pdf]

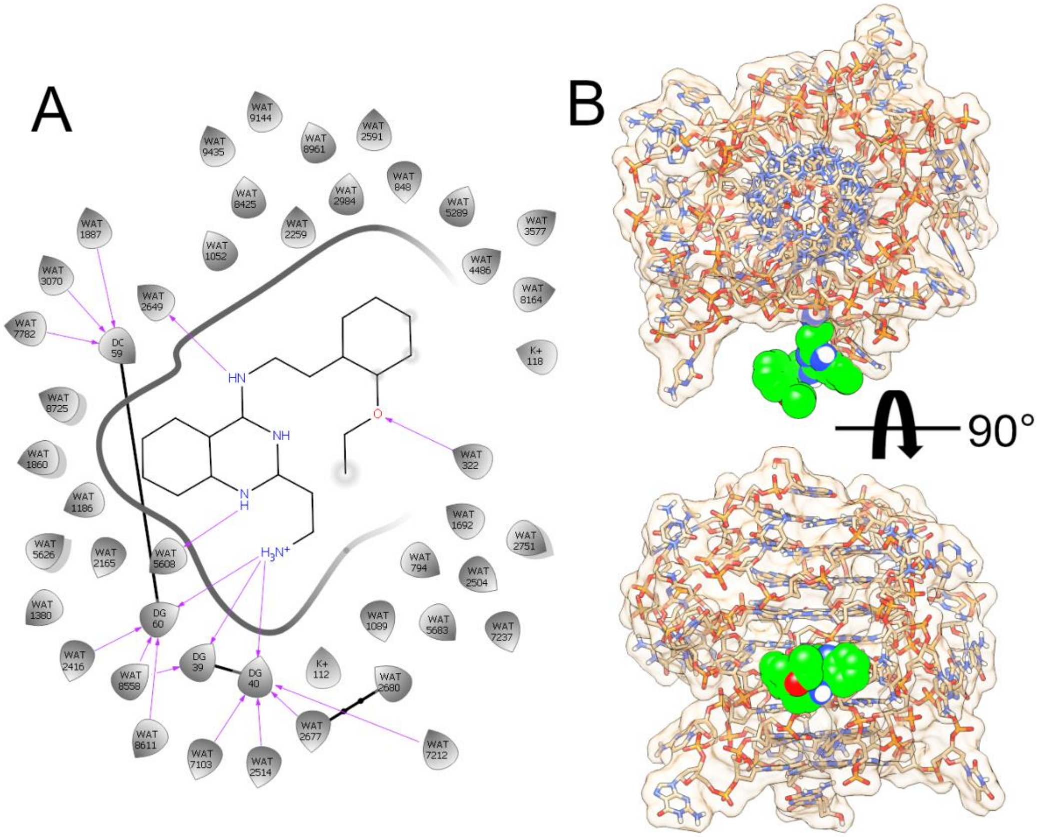

Figure S7. Interaction network and space-fill representation of 3B1 after 5ns of explicit solvent MD simulation at site DG38. (A) Hydrogen bonding network showing multiple water interactions, although none are coordinated with residues on the quadruplex. (B) Space-fill representation of hTERT-FL with 3B1 (green) shown as spherical representation. The two orientations emphasize how far the 3B1 ethoxy group (oxygen in red) extends away from the quadruplex.
