# Supplementary material for: Drug discovery of small molecules targeting the higher-order hTERT promoter G-quadruplex
Source: PLoS One. 2022 Jun 16;17(6):e0270165. doi: 10.1371/journal.pone.0270165 (PMC9202945; doi:10.1371/journal.pone.0270165)
Supplement: S8 Fig — A and C show averaged titrations of hTERT and 3B1 in the absence and presence of 250 μg/mL C.T., respectively. B and D show averaged titrations of hTERT and GTC365 in the absence and presence of 250 μg/mL C.T., respectively. Each curve was fit with a 1-site binding model in PALMIST v1.5.8 [60] using the cold fluorescence mode and the Kd values, 68.3% confidence intervals, and fits (rmsd) are given in the inset in blue. (PDF) [file pone.0270165.s008.pdf]

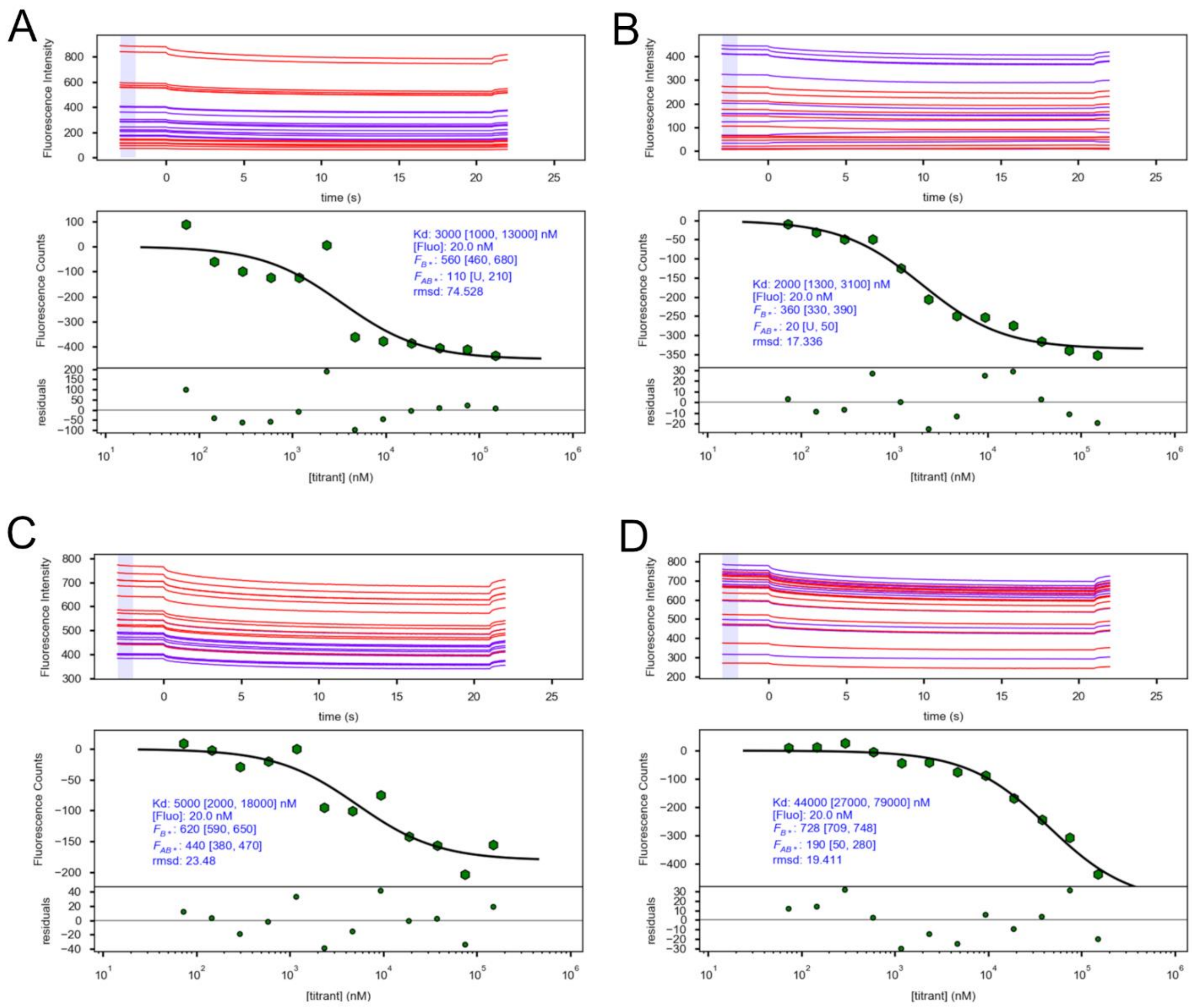

Figure S8. MST titrations of Cy5-hTERT-FL with 3B1 and GTC365 in the presence or absence of calf thymus (C.T.) DNA. A and C show averaged titrations of hTERT and 3B1 in the absence and presence of 250 μg/mL C.T., respectively. B and D show averaged titrations of hTERT and GTC365 in the absence and presence of 250 μg/mL C.T., respectively. Each curve was fit with a 1-site binding model in PALMIST v1.5.8 using the cold fluorescence mode and the K_d values, 68.3% confidence intervals, and fits (rmsd) are given in the inset in blue.
