# Supplementary material for: Drug discovery of small molecules targeting the higher-order hTERT promoter G-quadruplex
Source: PLoS One. 2022 Jun 16;17(6):e0270165. doi: 10.1371/journal.pone.0270165 (PMC9202945; doi:10.1371/journal.pone.0270165)
Supplement: S1 Raw images — (PDF) [file pone.0270165.s009.pdf]

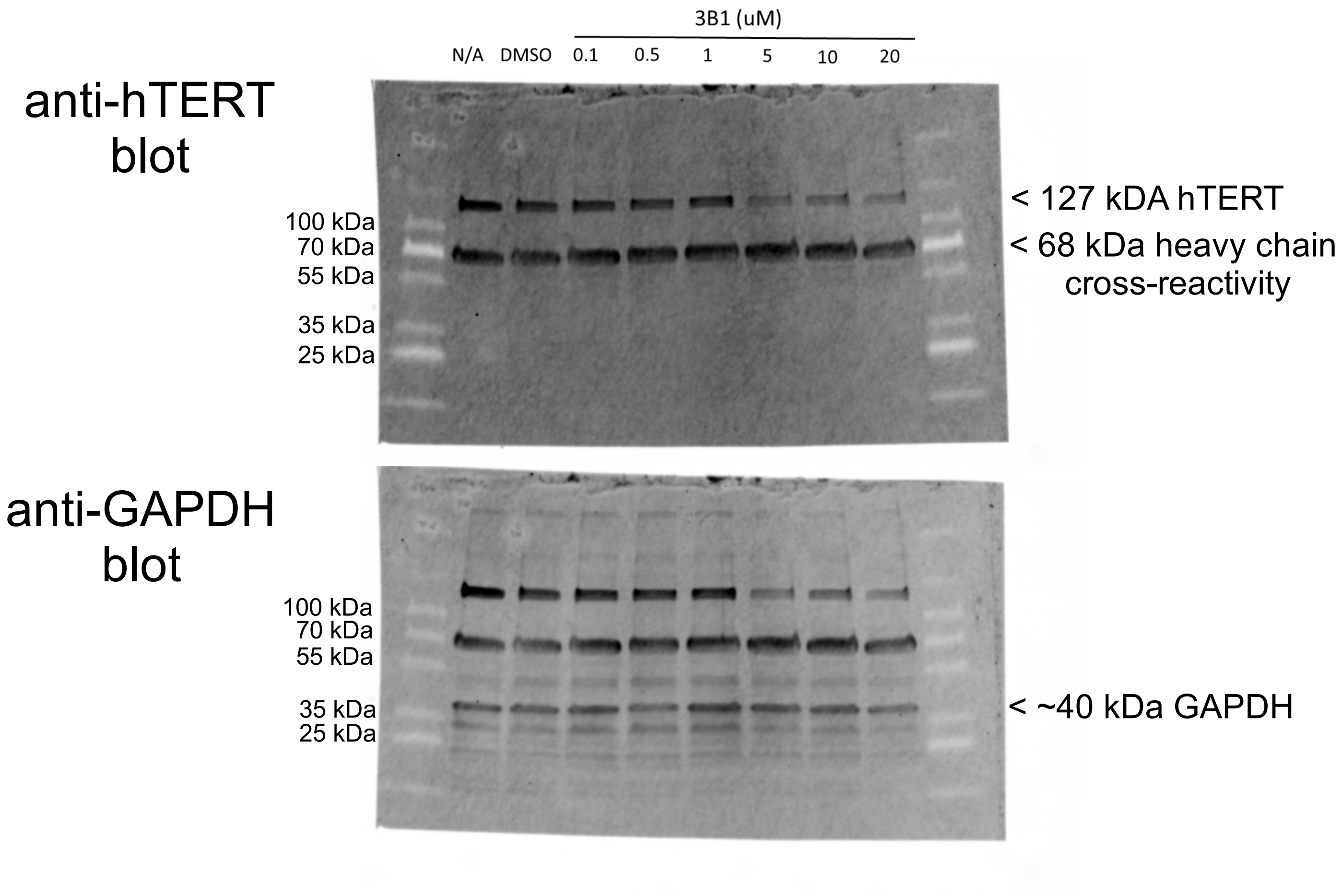

Background bands that are not assigned are from a protein cocktail antibody solution (targeting CdK2 and pTyr15). Ladder is the PageRuler Plus Protein Standard.
