# Supplementary material for: Drug discovery of small molecules targeting the higher-order hTERT promoter G-quadruplex
Source: PLoS One. 2022 Jun 16;17(6):e0270165. doi: 10.1371/journal.pone.0270165 (PMC9202945; doi:10.1371/journal.pone.0270165)
Supplement: Attachment — Submitted filename: Response to Reviewers.docx [file pone.0270165.s010.docx]

Fenfei Leng, Ph. D.
Academic Editor
PLOS ONE

May 18, 2022

Dear Dr. Fenfei Leng,

Thank you for your letter informing us of the PLOS ONE decision. We have uploaded the minor revision requested in the format that was requested. We have considered and addressed all comments by the reviewers and the editor:

***Reviewer 1 Comment:***

***… The manuscript is well written and scientifically very convincing. For these reasons the manuscript deserves to be published as it is.***

Response: No revisions were requested. We thank the reviewer for their enthusiastic support.

***Reviewer 2*** provided highly complimentary and positive comments. Only two comments were made that required action and revision. The two comments are addressed below.

***Reviewer 2 Comment:***

***The low rate of return of compounds with acceptable affinity for hTERT, however, raises a concern with the screening set. A better G4/nucleic acid set of molecules appears to be an important future area for development for G4 drug discovery.***

*Response:* Additional text added

“Although our rate of return of compounds with acceptable affinity for hTERT is low, we believe there are two main reasons why this may be. The first is that most virtual screening software scoring functions are parameterized for proteins and, second, even if parameterized for DNA the higher-order G4 binding pockets are not canonical DNA. This opens up an opportunity for improving scoring functions with more data from higher-order quadruplex:ligand complex structures.”

This was an excellent point to make and it really justifies more investigations such as ours as we need more validated higher-order DNA:ligand complexes to be able to parameterize such screening efforts to be more efficient. This is the first report of higher-order DNA:ligand screening effort, so therefore we could not parameterize the scoring functions as no structures exist.

***Reviewer 2 Comment:***

***Minor Point: The activity for 3B1 in cancer cells (Figure 6) seems relatively low, given its binding affinity and selectivity for hTERT. It would be useful for the authors to briefly address this point.***

*Response:* Additional text added

“Although 3B1 binds with moderate affinity and selectivity to the higher-order hTERT G4, its effect on hTERT production in cells was relatively mild (**Figure 6**). The first and most obvious reason is that 3B1, which has a privileged quinazoline moiety(83), has been sequestered by off-target proteins, either in the media, cell milieu, or both. Selectivity over proteins will be addressed in subsequent work aiming to improve on 3B1 as a lead molecule. Another reason, that is less well defined and incomparable to traditional protein or duplex targeting, is the temporal dynamics of promoter G4 structures(5). It is unclear how persistent the hTERT core promoter G4 is at any given time. However, both hTERT promoter activity and G4-formation are highest during S-phase(5, 84), which helps in explaining why 3B1 has only a modest effect after 72 hours.”

***Reviewer 3 Comment:***

***This is a well written solid manuscript discussing drug discovery for small molecules to target the hTERT sequence. A variety of experimental and computational studies were carried out. The results were presented in a logical systematic fashion and the conclusions drawn reasonable. I see no need for any revision.***

Response: No revisions were requested. We thank the reviewer for their enthusiastic support.

***Editor’s Comment:***

***Your article has been reviewed by three experts in the field. Please revise it according to comments by reviewer #2 and submit a revised version of the manuscript.***

Response: We have done so. Reviewer 2 had only two comments that required action as addressed above.
We have fully addressed all of Reviewer 2 comments.

We hope that you find the manuscript acceptable for publication with all the requested responses addressed.

The blot/gel image data are in Supporting Information

Sincerely,

John O. Trent, Ph.D.

Deputy Director, Basic and Translational Research, UofL Health Brown Cancer Center

Wendell Cherry Endowed Chair in Cancer Translational Research

Leader, UofL Health Brown Cancer Experimental Therapeutics Program

Professor of Medicine, Biochemistry and Molecular Genetics

Fellow, National Academy of Inventors

Suite 224, Clinical Translational Research Building,

Email: john.trent@louisville.edu
